# Platelet Reactivity and Fibrin Clot-Strength as assessed by TEG in Patients with Atrial Fibrillation undergoing Percutaneous Coronary Intervention

**DOI:** 10.1007/s12265-025-10673-4

**Published:** 2025-08-27

**Authors:** Diona Gjermeni, Hannah Vetter, Sofia Szabó, Viktoria Anfang, Carina Juelch, Stefan Leggewie, David Hesselbarth, Markus Jäckel, Daniel Duerschmied, Dietmar Trenk, Dirk Westermann, Christoph B. Olivier

**Affiliations:** 1 Department of Cardiology and Angiology, Faculty of Medicine, University Heart Center Freiburg - Bad Krozingen, University of Freiburg, Hugstetter Str. 55, 79106 Freiburg, Germany; 2 Department of Cardiology, Haemostaseology and Medical Intensive Care, Medical Center Mannheim, Medical Faculty Mannheim, Heidelberg University, Heidelberg, Germany; 3 European Center for AngioScience (ECAS) and German Center for Cardiovascular Research (DZHK) Partner Site Heidelberg/Mannheim, Mannheim, Germany

**Keywords:** Atrial fibrillation, Percutaneous coronary intervention, Platelet aggregation, Thromboelastography

## Abstract

Platelet reactivity (PR) in clopidogrel-treated patients undergoing percutaneous coronary syndrome (PCI) associates with ischemic and bleeding risk. The aim was to investigate the association of PR and global hemostasis with this risk in patients with atrial fibrillation (AF) undergoing PCI. TEG was performed on day 1–3 after PCI. 168 patients were included. Mean age was 79 years (IQR 72–82). 101 (60%) patients had high platelet reactivity (HPR). HPR was not associated with the composite outcome of MACE (HR 1.23 [ CI 95% 0.43–3.49], *p* = 0.700). 33(19.6%) patients had HPR and increased platelet–fibrin clot strength and showed a trend for association with higher ischemic risk (HR 2.83 [CI 95% 0.70–8.06], *p* = 0.078). Rates of HPR in patients with AF undergoing PCI were high. Neither HPR nor LPR predicted ischemic or bleeding risks. Patients with HPR and increased platelet–fibrin clot strength may be at higher risk for ischemic events.

**Graphical Abstract:**

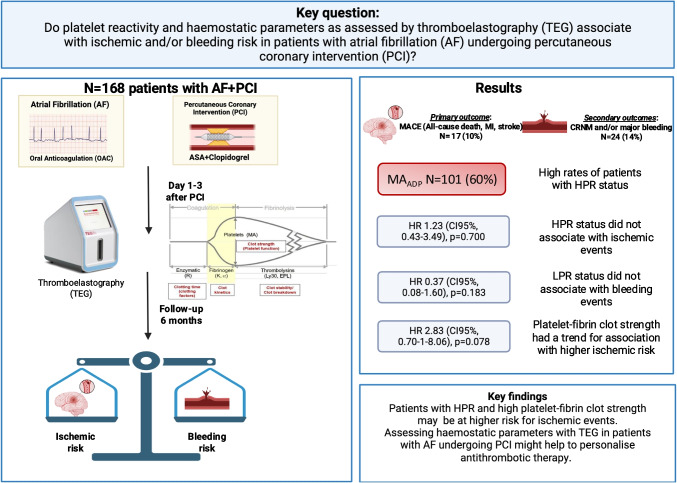

**Supplementary Information:**

The online version contains supplementary material available at 10.1007/s12265-025-10673-4.

## Introduction

To avoid ischemic complications such as stent thrombosis patients undergoing percutaneous coronary intervention (PCI) are treated with P2Y12-inhibitor [1]. Among patients with atrial fibrillation (AF), > 50% present with concomitant coronary artery disease (CAD) [2]. Guidelines recommend treatment with a direct oral anticoagulant (DOAC), a P2Y12 inhibitor (preferably clopidogrel), and acetylsalicylic acid (ASA) for the first week post PCI. Afterwards, ASA [3] is omitted in these patients [4, 5] and they are treated with dual antithrombotic therapy consisting of clopidogrel and a DOAC posing patients with altered platelet reactivity (PR) at risk for complications. Different platelet function testing (PFT) methods are used to determine high and low platelet reactivity statuses (HPR and LPR), which have both been linked to adverse events in patients with CAD undergoing PCI [6–9]. HPR status was relatively uncommon in patients who received clopidogrel loading dose prior to PCI [10] when assessing PR by thromboelastography (TEG). Nevertheless, other studies reported that 50–51% of the patients undergoing PCI present with HPR status [11, 12]. Additionally, elevated platelet–fibrin clot strength, assessed by TEG, was associated with a higher risk for major adverse cardiac events (MACE) [11] suggesting that it could be a useful marker to detect ischemic risk.

This study aimed to evaluate the association of HPR status and platelet–fibrin clot strength as assessed by TEG, with adverse events in patients with AF treated with an oral anticoagulation (OAC) undergoing PCI.

## Methods

### Study Design and Population

This was an observational prospective cohort study. 171 Participants were enrolled between November 2020 and October 2022 at the Department of Cardiology and Angiology (formerly Departments of Cardiology and Angiology I and II, respectively) at the University Heart Center Freiburg-Bad Krozingen. The protocol and amendments were approved by the local ethics committee, privacy and security offices, and institutional review board (Ethic protocol number: 21–1146). The study was registered at the German register for clinical trials (DRKS00024509). Patients with AF, indication for OAC according to the guidelines, new coronary stent implantation, and treatment with clopidogrel were enrolled. Detailed inclusion and exclusion criteria are listed in Table S1, supplement. Because of technical failure during TEG measurement one patient was excluded from the study. Two patients were excluded because peri-interventational medication was changed from the interventional cardiologist and there was divergence from inclusion criterias.

Sample size was calculated using Cox-proportional hazards framework. Since'time-to-event'methods were developed as'survival'methods, the primary parameter of interest is called the hazard ratio (HR). The hazard is the probability of the event occurring in the next instant given that it hasn't yet occurred. The HR is then the ratio of the hazards between two groups. We expected to detect HPR in 40% of the patients. In patients undergoing coronary stent implantation (irrespective of AF) with HPR status Gurbel et al. [6] reported a HR of 10.3 (95% confidence interval [CI], 5.6–21.3) for a composite ischemic outcome. For this study, involving patients with additional AF, we conservatively estimate this increased risk according to the lower bound of the reported 95% CI with 5.6. Assuming an event rate of approximately 7% at 6 months [13], 158 participants needed to be analyzed (two-sided alpha: 5%; beta: 20%) to detect such association. Considering a drop-out rate of 5%, 166 participants needed to be enrolled.

### Coronary Intervention and Patient Management

All patients were treated with at least one stent according to the operator discretion. After arterial puncture and introduction of sheaths all patients received initially heparin i.v. 70–100 U/kg per standard of care. Further heparin dose administrations were adjusted according to the activated clotting time (ACT). All patients were treated with OAC and clopidogrel (loading dose of 300 and 600 mg). Inclusion in the study was performed from day 1–3 after PCI. Duration of the atithrombotic treatment was left at the discretion of the interventionalist cardiologist after considering the individual bleeding and ischemic risk. Baseline characteristics, medical history, procedural data, and medication at discharge were collected per patient interview or medical records. The primary outcome consisted of major adverse cardiac events (all-cause death, myocardial infarction or stroke) at 6 months ± 2 weeks [14]. Secondary outcome consisted of major bleedings or non-major clinically relevant bleeding (NMCR) according to the International Society on Thrombosis and Haemostatis (ISTH) [15]. Follow-up was performed by structured telephone interview. Two independent physician reviewers blinded to TEG and laboratory values adjudicated all clinical outcome events including self-reported minor bleedings. Major discrepancies were resolved by the principal investigator (CBO) who was also blinded to the TEG results.

### Hemostatic and Platelet Function Measurements

Blood was drawn on day 1–3 after PCI and all patients received clopidogrel 1-2 h before blood draw as per study protocol. On the day of planed blood draw all patients were under maintenance doses of clopidogrel. Clopidogrel loading doses was given on the day of index PCI if the patient was naïve. For platelet functioning assay, 17 IU/mL Li-heparin (Becton, Dickinson and Company, Heidelberg, Germany) monovettes were used. Citrated blood monovettes (S-Monovette, 9NC: 0.106 mol/L citrat) were used for global hemostasis. Blood samples were stored at room temperature and the measurements were performed after 30 min and within 4 h form blood draw. Thromboelastography was performed with TEG6s Hemostasis Analyzer (Haemonetics Corp., Boston, MA, USA). Two multichannel cartridges holding dried reagents were used separately, one for assessing platelet function and the other for the global hemostasis. 400 μL blood were automatically aspirated into the testing chamber in each cartridge and mixed with the required reagents. 2 μM ADP was used as a reagent for evaluating platelet function. Kaolin with heparinase (concentration > 1800 IU/mL), a neutralizer of the effects of heparin, were used for the evaluation of platelet–fibrin clot strength. Platelet-fbirin clot strength is an indicator of the overall aggregability in this patient cohort. Afterward, blood is automatically exposed to ultrasound pulses (20–500 Hz frequency) that change during coagulation depending on clot strength. At this stage, TEG begins to measure clot formation in real-time.The device measures the viscoelastic properties of the blood as it clots, providing detailed information about clot initiation, strength, and stability, which are automatically displayed as graphical traces and as numerical values. The maximum amplitude (MA, [mm]) describes the maximum clot strength. HPR status was defined as a MA_ADP_ ≥ 47 mm after stimulation with ADP, as suggested from the manufacturer based on the updated expert consensus, whereas MA_ADP_ ≤ 31 mm was defined as LPR status [16].

Kaolin-activated channel HKH-channel was used for determining MA_Thrombin_ (reference values per manufacturer: 53–68 mm) [16]. MA_Thrombin_ quantifies the changes in fibrinogen and clot strength [11]. We then analyzed the presence of both HPR status as well as increased MA_Thrombin_ (increased platelet–fibrin clot strength) as a separate subgroup [11]. Activation of the thromboxane pathway using arachidonic acid (ASA) reflected the platelet contribution to clot strength (MA_ASA_). Additionally, the percentage of aggregated platelets after ADP stimulation (%-Agg. ADP) was determined. The reference values varied between 83–100%. Furthermore, MA_CFF_ representing fibrin contribution to clot strength (cut –off 15–32 mm), as well as MA_CRT_, representing platelet–fibrin contribution to the overall clot strength (cut-off 52–70 mm) were determined.

CRT-ly30 measures the percentage of clot breakdown that occurs within 30 min after the TEG tracing reaches its maximum strength. CK-R refers to the citrated kaolin reaction time, which is a measure of the time it takes for blood to begin clotting when stimulated by kaolin. This parameter is used to assess the initiation phase of coagulation, indicating the efficiency of the coagulation cascade. Lastly, R time represents the time it takes for fibrin clot formation to begin and correlates with the coagulation parameter of activated partial thromboplastin time (aPTT) [17].

### Statistical Considerations

Results are presented as absolute numbers with percentages for binomial variables and as medians with interquartile ranges for continuous variables. Data were compared with Chi-quadrat Test for categorical variables and continuous variables were compared with a 2-sided unpaired t-test or Mann–Whitney U test. Pearson corellation was performed to evalute the association of laboratory parameters with TEG parameters.

To evaluate the association of different paramters, with the ischemic and bleeding outcomes, as well as with TEG paramters, univariable and multivariable linear and logistic regression were performed and odds ratios (ORs) with confidence interval (CI) of 95% were calculated. To calculate cumulative event rates in time, Kaplan-Maier curves were generated and log-rank tests were performed. Furthermore, Cox-proportional Hazard ratio test was performed and HR with 95% CI were calculated. All tests were two tailed and a p-value of 0.05 or less was considered significant. Data were analyzed with Prism 12.0.13 (GraphPad Software, La Jolla, CA, USA) and SPSS 29.0.0.0 (SPSS Inc., Chicago, IL, USA).

## Results

### Patient Population and Peri-Procedural Medication

A total of 168 patients undergoing PCI with AF were included in the study. Median age was 79 (interquartile range, IQR 72–83) years and 123 (73%) patients were male. Median CHA_2_DS_2_-VASC Score was 5 (IQR 4–6). Comorbidities were present as follows: arterial hypertension in 149 (89%) patients, hyperlipidemia in 118 (70%) patients and renal impairment in 72 (43%) patients. 54 (32%) participants underwent PCI due to acute coronary syndrome (ACS). 89 (53%) patients were treated with two or more stents during PCI (Table 1).

**Table 1:** Clinical baseline characteristics

Baseline Characteristics	Total *N* = 168	
Demographics		
Age, years	79	(72–83)
Male	123	(73%)
BMI, kg/m^2^	26	(25–30)
Medical History		
Type of atrial fibrillation		
Paroxysmal	105	(63%)
Persistent	39	(23%)
Permanent	24	(14%)
CHA_2_DS_2_–VASc score	5	(4–6)
HAS–BLED score	3	(3–4)
Hypertension	149	(89%)
Diabetes	59	(35%)
Hyperlipidemia	118	(70%)
Heart failure	54	(32%)
Renal impairment	72	(43%)
Nicotine abuse	56	(33%)
GI–/intracranial bleeding	16	(9%)
Prior stroke/TIA	28	(17%)
Prior PCI	80	(48%)
Prior myocardial infarction	36	(21%)
Family history of CAD	39	(23%)
Periprocedural		
Indication for PCI		
ACS	54	(32%)
Elective	114	(68%)
Single vessel disease	27	(16%)
Left main disease	52	(31%)
Implanted stents		
1	79	(47%)
2–4	81	(48%)
≥ 5	8	(5%)
Thrombocyte count, × 10^3^/μL	211	(174–272)
Fibrinogen, mg/dl	413	(343–465)
IPF in %	3.8	(2.7–5.5)

Values are n (%) or median (interquartile range). Abbreviations: ACS, acute coronary syndrome; BMI, body mass index; CAD, coronary artery disease; GI–bleeding, gastrointestinal bleeding; IPF, immature platelet fraction; PCI, percutaneous coronary intervention; TIA, transient ischemic attack

All patients were treated peri-procedurally with ASA and 167 (99%) with clopidogrel. 133 (79%) patients received loading doses of clopidogrel, while 34 (20%) were already on maintenance doses. 138 (82%) patients were treated with an OAC at the time of inclusion in the study. Peri-procedurally, 157 (93%) patients were treated with ASA. At discharge, all patients had clopidogrel therapy and OAC. Oral anticoagulation was stopped in one patient because of bleeding complications at the time of discharge. At discharge, 7 (4%) patients were treated with a vitamin K antagonist whereas, all others were prescribed a DOAC. Rates of DOAC prescription at discharge are as follows: rivaroxaban 59 (35%), apixaban 59 (35%), edoxaban 37 (22%), and dabigatran 5 (3%). Detailed information regarding peri-procedural medication, medication at discharge, as well as medication at follow-up is shown in Table S2, supplement.

### Clinical Outcomes and Prognostic Implications of Global Platelet Aggregation

**Fig. 1:**
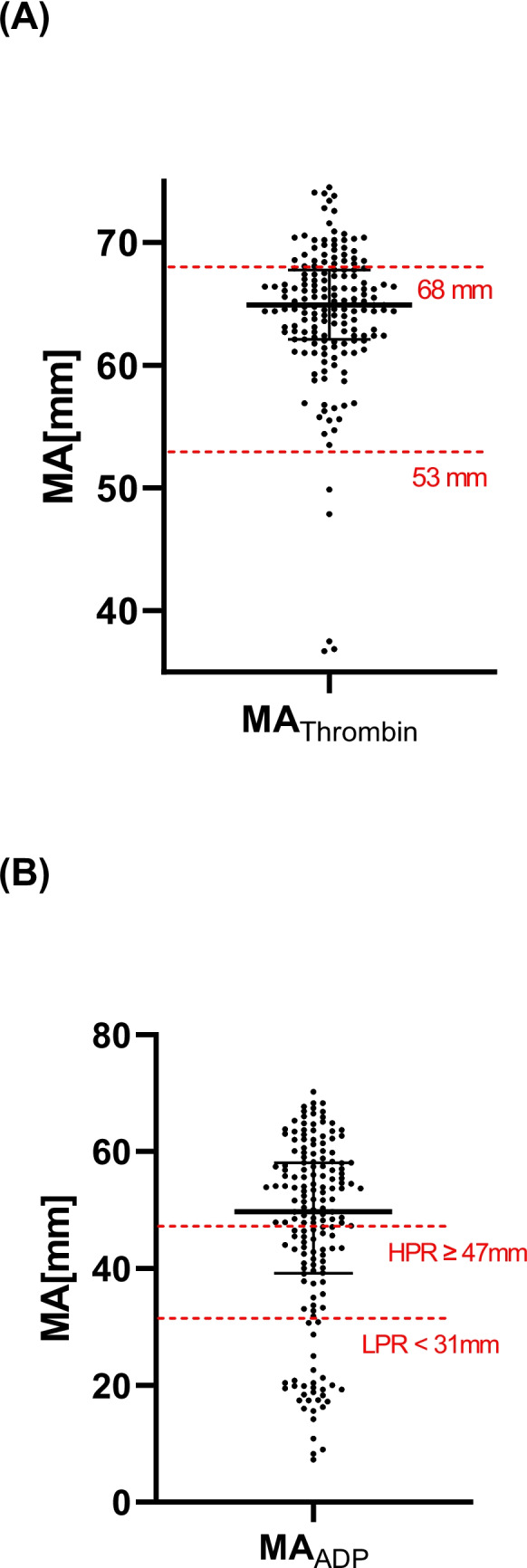
Platelet reactivity of patients with AF undergoing PCI represented as values of (**A**) MA_Thrombin_ and (**B**) MA_ADP_. Black lines represent Median and interquartile range. Red lines indicate conventional cut-off values with interquartile range (MA_ADP_ ≥ 47 mm for HPR and MA_ADP_ < 53 mm) or reference values suggested by the manufacturer (MA_Thrombin_ 53–68 mm), respectively (**A**)

While high on-clopidogrel PR (MA_ADP_ ≥ 47 mm) was present in 101 (60%) patients, 31 (18%) patients had low on clopidogrel PR after PCI (Table 2, Fig. 1). Median of the percentage of platelet aggregation after stimulation with ADP (%-aggr. ADP) was 72% (IQR 48%−85%) and 46 (27%) patients had values of %-aggr. ADP within the reference range (Figure S2, supplement). Platelet aggregation median values under ASA distributed mostly below the lower reference value (Figure S2, supplement).

**Table 2:** Primary and secondary outcomes at 6 months ± 2 weeks follow-up

Outcomes	Total *n* = 168		HPR *n* = 101 (60%)	No HPR/no LPR *n* = 36 (22%)	LPR *n* = 31 (18%)	*p*-Value
Primary outcome						
Major adverse cardiac events	17	(10%)	10 (10%)	3 (8%)	4 (13%)	0.820
Death	10	(6%)	7 (7%)	2 (6%)	1 (3%)	0.743
Myocardial infarction	5	(3%)	2 (2%)	1 (3%)	2 (6%)	0.439
Stroke	2	(1%)	1 (1%)	0 (0%)	1 (3%)	0.458
Secondary outcomes						
NMCR or major	24	(14%)	16 (16%)	5 (14%)	3 (10%)	0.690
NMCR	9	(5%)	7 (7%)	1 (3%)	1 (3%)	0.840
Major	15	(9%)	9 (9%)	3 (8%)	3 (10%)	0.982
Any bleedings	72	(43%)	43 (43%)	15 (42%)	14 (45%)	0.852
Minor bleedings	48	(29%)	27 (27%)	10 (28%)	11 (35%)	0.611

The values are in number and percentage, n (%). Abbreviations: NMCR non-major clinically relevant, LPR low platelet reactivity, HPR high platelet reactivity

The primary composite outcome occurred in 17 (10%) patients at 6 months ± 2 weeks follow-up (Table 3). Stroke and myocardial infarction (MI) event rates were low with 5 (3%) and 2 (1%), respectively. Secondary bleeding outcome occurred in 24 (14%) patients. Of these events, 15 (9%) were major bleedings and 9 (5%) were NMCR bleedings. Minor bleedings occurred in 48 (29%) patients and any bleeding in 72 (43%) patients (Table 2).

**Table 3:** Primary and secondary outcomes at 6 months ± 2 weeks follow-up for patients with HPR vs. no HPR, as well as for patients with LPR vs. no LPR. Cox-regression analysis for the association of HPR with the primary ischemic outcome and LPR with the secondary bleeding outcome, respectively

Outcomes	Total *n* = 168		HPR *n* = 101 (60%)	No HPR *n* = 67 (40%)	*p*-value	LPR *n* = 31 (18%)	No LPR *n* = 137 (82%)	*p*-value	HR	CI 95%	*p*-value
Primary outcomes									**HPR**		
Major adverse cardiac events	17	(10%)	10 (10%)	7 (10%)	0.908	4 (13%)	13 (9%)	0.569	1.228	0.433–3.486	0.700
Death	10	(6%)	7 (7%)	3 (4%)	0.511	1(3%)	9 (7%)	0.477	0.716	0.152–3.373	0.673
Myocardial infarction	5	(3%)	2 (2%)	3 (4%)	0.351	2 (6%)	3 (2%)	0.207	1.956	0.327–11.705	0.462
Stroke	2	(1%)	1 (1%)	1 (1%)	0.760	1 (3%)	1 (1%)	0.235	2.918	0.182–46.651	0.449
Secondary outcomes									**LPR**		
NMCR or major	24	(14%)	16 (16%)	8 (12%)	0.479	3 (10%)	21 (15%)	0.159	0.367	0.084–1.605	0.183
NMCR	9	(5%)	7 (7%)	2 (3%)	0.680	1 (3%)	8 (6%)	0.764	1.137	0.235–5.498	0.873
Major	15	(9%)	9 (9%)	6 (9%)	0.992	3 (19%)	21 (15%)	0.657	0.458	0.056–3.766	0.468
Any bleedings	72	(43%)	43 (43%)	29 (43%)	0.820	14 (45%)	58 (42%)	0.914			
Minor bleedings	48	(29%)	27 (27%)	21 (31%)	0.765	11 (27%)	37 (27%)	0.301			

The values are in number and percentage, n (%). Abbreviations: NMCR non-major clinically relevant, HPR high platelet reactivity, HR hazard ratio, CI confidence interval

Kaplan–Meier curves for time to the primary composite outcome of MACE and secondary bleeding outcome are represented in (Figure S1, supplement).

HPR status was not associated with the primary composite outcome. MACE occurred with similar rates (10%) in both groups of patients with HPR status and the ones without (HR for HPR 1.23 [CI 95% 0.43–3.49], *p* = 0.700) (Table 3). Similarly, LPR status was not associated with the secondary bleeding outcome (HR 0.37 [CI 95% 0.08–1.60], *p* = 0.183) (Table 3). There were no differences in the distribution of event rates when considering the singular bleeding complications separately, between patients with LPR status and those without (Table 3). Additionally, HPR status was not a protective factor for bleeding complications. NMCR or major bleedings occurred in 16 (16%) patients presenting with HPR vs. 8 (12%) patients without HPR, *p* = 0.479 (Table 3).

When defining HPR status according to the %-agg. ADP, no significant differences in the rates of myocardial infarctions (4% vs. 2%) or strokes (2% vs. 1%) were observed for patients presenting with HPR compared to patients with PR within the reference range (Table S3, supplement).

### Prognostic Implications of Global Hemostasis in the Clinical Outcomes

Overall thrombus formation was measured with the hemostasis assay through different parameters. MA_Thrombin_ median was 64.9 mm (IQR 62 mm-68 mm). A total of 38 (23%) patients had increased MA_Thrombin_, indicating an increased platelet–fibrin activity contributing to clot strength, whereas in 5 (3%) patients MA_Thrombin_ was reduced (Fig. 1). The median of MA_CRT,_ representing clot strength, was 66 mm (IQR 63 mm-69 mm) and was within the normal range for the majority of patients (Figure S3, supplement). MA_CFF_ representing fibrin contribution to the thrombus formation was in 44 (26%) of the patient increased (Figure S3, supplement). Other haemostatic parameters that were assessed by TEG are represented in the Figure S3, supplement. Of note, CK-R values representing the duration of initiation phase of coagulation cascade was mostly within refenrence values.

**Fig. 2:**
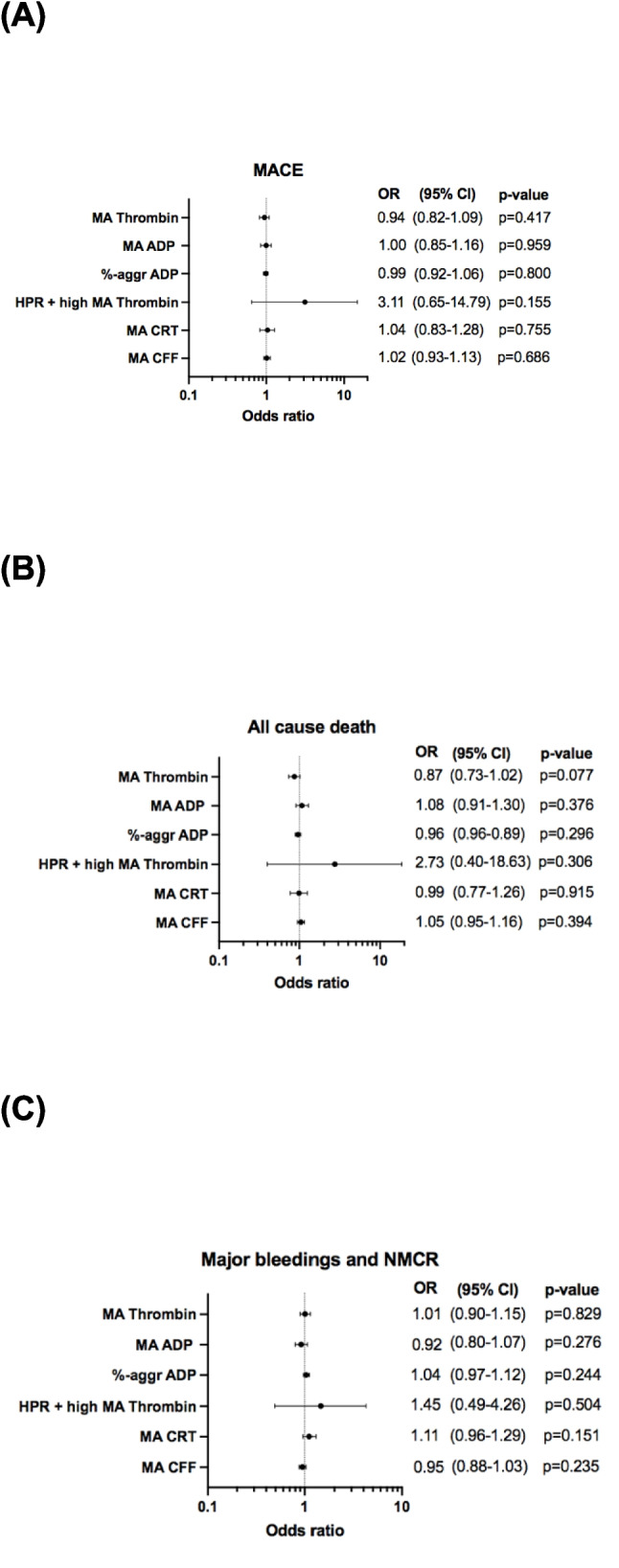
Association of different parameters measured by TEG representative of platelet and hemostasis mapping with the (**A**) primary composite ischemic outcome of MACE, (**B**) all-cause death, and (**C**) the secondary bleeding outcome at 6 months ± 2 weeks follow-up

To assess the prognostic implication of the multiple values, assessed by TEG a multivariable logistic regression analysis was performed (Fig. 2). MA_Thrombin_ was not associated neither with the primary composite outcome (OR 0.94 [CI 95% 0.82–1.09], *p* = 0.417), nor with the secondary bleeding outcome (OR 1.01 [CI 95% 0.90–1.15], *p* = 0.829) (Fig. 2). Additionally, MA_CRT_ and MA_CFF_ were not associated with both of the clinical outcomes.

A total of 33 (19.6%) patients presented with both HPR status and an increased MA_Thrombin_. In patients with increased platelet–fibrin clot strength and HPR status, the odds for the occurrence of composite ischemic outcome were 3.11 ([CI 95% 0.65–14.79]; *p* = 0.155) and for all-cause mortality were 2.73 ([CI 95% 0.40–18.63], *p* = 0.306). None of the other parameters measured with TEG was associated with the secondary bleeding outcome (Fig. 2).

**Fig. 3:**
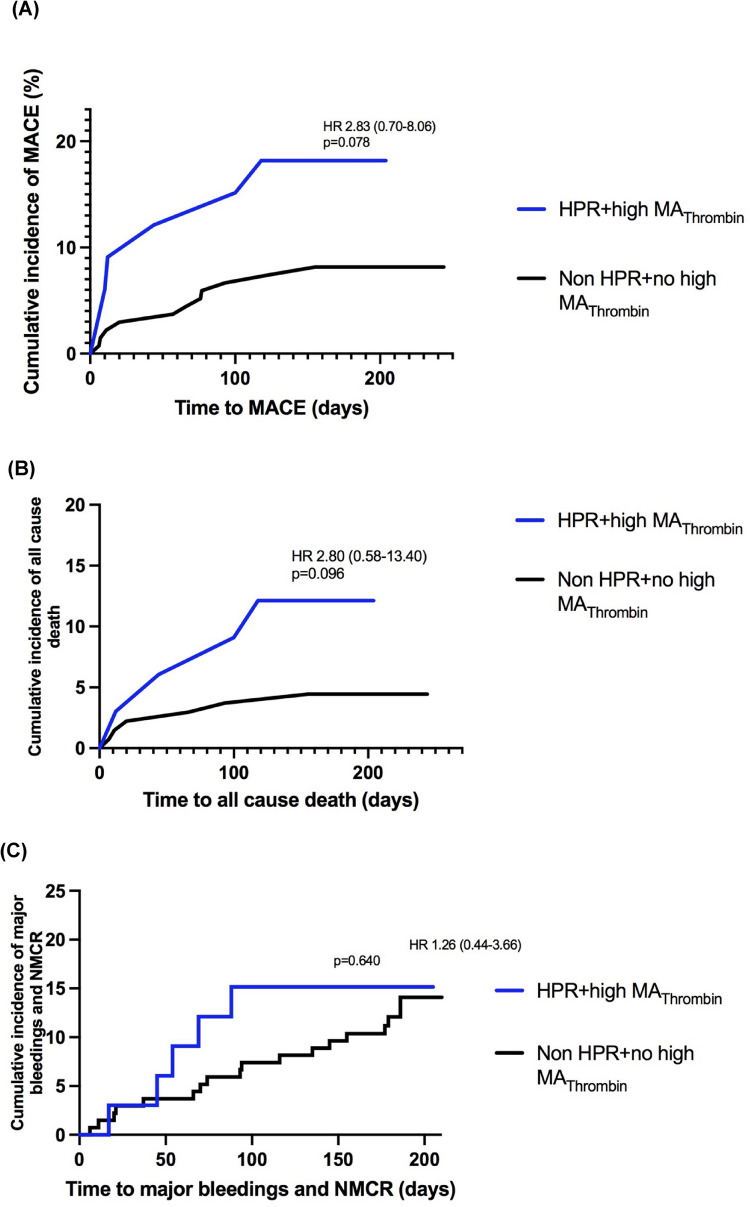
Kaplan-Maier-curves for (**A**) the comoposite primary ischemic outcome of MACE, (**B**) all cause death, and (**C**) major bleeding or NMCR for patients with HPR and increased platelet–fibrin clot-strength vs. rest

When comparing patients with HPR status and increased MA_Thrombin_ to the rest of the patients, there was a trend for an increased risk of the primary composite ischemic endpoint (HR 2.83 [CI 95% 0.70–8.06], *p* = 0.078) (Fig. 3).

**Fig. 4:**
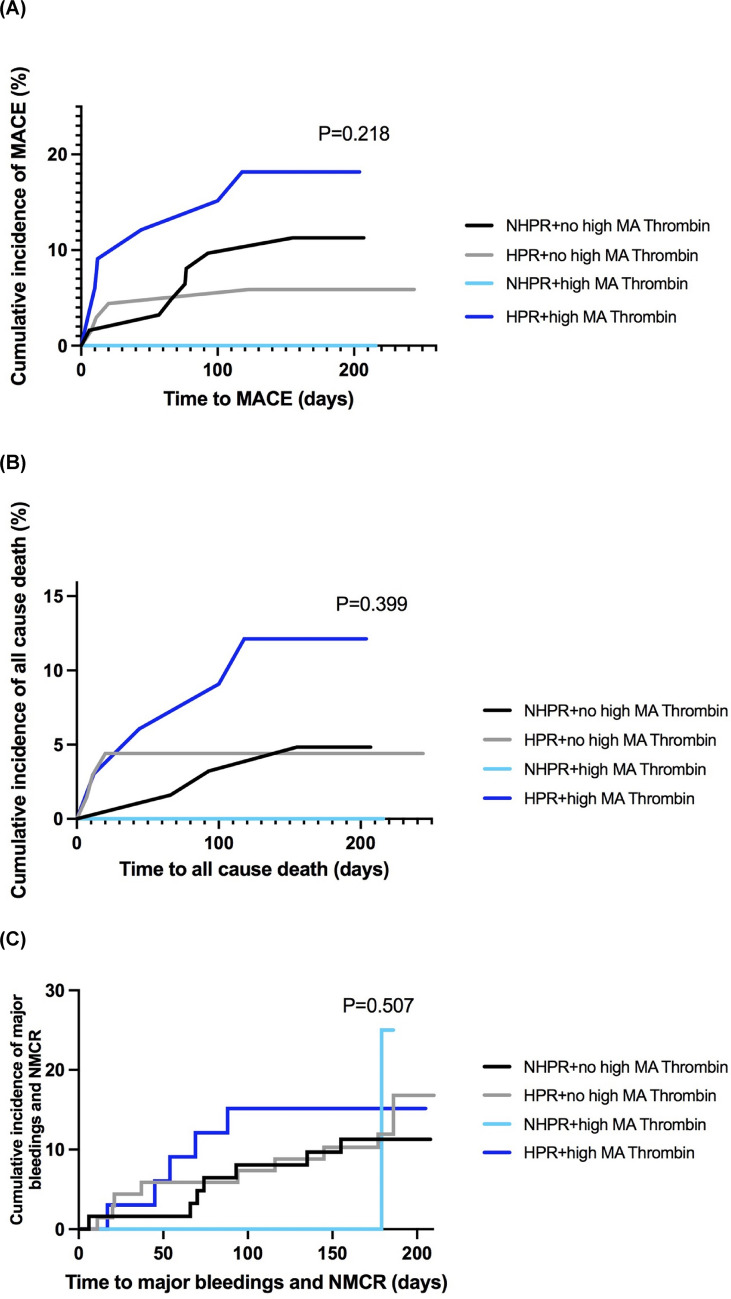
Kaplan-Maier-curves for primary ischemic outcome of (**A**) MACE, (**B**) all cause death, and (**C**) secondary outcome consisting of major bleeding or NMCR for patients grouped according to presence of HPR and increased or not platelet–fibrin clot-strength based on the suggested cut-offs from the manufacturer

When dividing patients in 4 groups: (1) both HPR and increased MA_Thrombin_, (2) no HPR status (NHPR) and MA_Thrombin_ not increased, as well as only (3) either increased MA_Thrombin_ or (4) HPR status, no significant difference between various patterns of platelet aggregation and clot strength status was detected for the occurrence of primary composite ischemic outcome (*p* = 0.218) and all cause death (*p* = 0.399) (Fig. 4).

### Factors Associated with TEG and Clinical Outcomes

To evaluate the association of baseline characteristics and laboratory biomarkers with TEG measurements a multivariable linear regression analysis was performed. Increase of age by one year and male sex were significantly associated with slightly higher MA_Thrombin_ (Table S4, supplement). Furthermore, if PCI was performed in an ACS setting, MA_ADP_ increased with 5.92 mm (95%CI 0.49–11.35, *p* = 0.033), while MA_Thrombin_ increased with 2.22 mm (95%CI 0.28–4.15, *p* = 0.025). MA_Thrombin_ was higher by 2.85 mm (95%CI 0.72–4.98, *p* = 0.009) when OAC was paused peri-interventionally (Table S4, supplement).

Platelet reactivity, when determined by MA_ADP_, was significantly associated with the clinical context and OAC therapy. The median MA_ADP_ value was increased among patients undergoing PCI for ACS compared to those undergoing elective PCI (54 mm vs. 48 mm, *p* < 0.001) (Figure S5A, supplement). Additionally, MA_ADP_ values were higher in patients whose OAC was paused on the day of PCI (50 mm) compared with continued OAC therapy (41 mm, *p* < 0.001) (Figure S5B, supplement).

Medians of platelet mapping parameters like MA_Thrombin_ and MA_ADP_ were higher when TEG was performed on day 3 after PCI (Figure S4, supplement) but only 13 (7%) patients underwent blood sampling at this delayed post-procedure time point. This limits the strength of any conclusion drawn by this grouping.

Median values of coagulation parameters such as fibrinogen as well as the absolute platelet count were within the normal range in this patient cohort (Table 1). A robust correlation between MA_Thrombin_ and MA_ADP_ with the coagulation parameters was evident (Figure S6, supplement). This findings suggests that platelet count and fibrinogen levels contribute both to platelet reactivity and thrombus strength as measured by TEG.

Lastly, MA_ADP_ did not correlate with R time when used a surrogate parameter for activated partial thromboplastin time (aPTT) [17] (Figure S7, supplement).

Neither ACS as indication for index PCI (OR 1.55, [95%CI 0.53–4.29], *p* = 0.40), nor the interruption of OAC peri-procedurally (OR 1.34, [95%CI 0.40–4.30], *p* = 0.63) associated with the composite ischemic outcome.

Furthermore, we incorporated baseline and procedural characteristics in a multivariable regression analysis. The presence of chronic kidney disease (OR 0.14, 95%CI 0.19–1.09, *p* = 0.061) or type of atrial fibrillation (OR 0.74, [95%CI 0.27–2.00], *p* = 0.56) were not significantly associated with the occurrence of MACE events at 6 months follow-up. When considering the secondary bleeding outcome, none of the variables included in the multivariable analysis were significantly associated. Despite slightly differences in the measured TEG parameters, as elucidated in the previous paragraph, regression analysis suggests that overall proceducal variables and pre-existing medical conditions and well as treatment with an OAC were not relevant predictors of both clinical outcomes.

## Discussion

This is the first study to evaluate the relationship between platelet reactivity and global hemostasis as assessed by TEG with clinical outcomes in patients with AF undergoing PCI, treated with clopidogrel and OAC. The findings indicate (1) a high prevalence of HPR in this patient cohort, though (2) HPR was not significantly associated with the primary composite ischemic outcome at 6 months and LPR did not associate with an increased risk of bleeding complications. (3) Patients with both HPR and increased platelet–fibrin clot strength may have a higher risk of ischemic events, highlighting the potential relevance of combined platelet aggregation and clot strength metrics for risk stratification in this specific patient cohort.

### Impact of Platelet Aggregation and Hemostatic Assays

In patients with AF undergoing PCI, ASA is omitted after one week from PCI and patients are treated with dual antithrombotic therapy (clopidogrel and an OAC) leading to a potentially increased ischemic risk for patients presenting with HPR status. Differences of platelet aggregability might help to identify patients at risk for complications. In contrast with our findings, previous studies including patients undergoing PCI but treated with clopidogrel without AF, have reported lower rates of HPR status when performing TEG. The median MA_ADP_ in the current patient cohort was significantly higher (51 mm in a previous study vs. 68 mm in our patient cohort) [6]. Another study showed that HPR rates were higher when PR was assessed with TEG as compared with multiple electrode aggregometry (MEA) [18]. Similarly in our study, HPR rates were 60% when considering the cut-off for MA_ADP_ suggested from the manufacturer which is based on the updated expert consensus (suggested cut-off MA_ADP_ ≥ 47 mm) [16]. One influencing factor might be that other studies did not always assessed HPR with TEG6s [11, 12], and a variability depending on the specific TEG device such as TEGS 5000 might be present.

Growing evidence suggests an increasing role of hypercoagulability represented by the clot-strength assessed by TEG in the occurrence of ischemic events in patients undergoing PCI. Increased clot-strength is an important factor for thrombotic events in patients with normal platelet aggregation status [6, 11, 19, 20]. Platelet–fibrin clot strength represented by MA_Thrombin_ (median 64.9 mm) in this study was more similar to the median of MA_Thrombin_ in post-PCI patients without ischemic events (65 mm) [20].

Rates of increased platelet–fibrin clot strength in this patient cohort were slightly lower when compared to a large cohort of 2,512 patients undergoing PCI (MA_Thrombin_ 23% vs. 39%). Additionally, MA_Thrombin_ was increased in 40% of the patients with peripheral arterial disease [21]. When comparing trauma patients treated with rivaroxaban with the ones without oral anticoagulation, MA_Thrombin_ median as assessed by TEG6s assay, was low and similar to the patients included in our study [22]. Similarly, lower MA_Thrombin_ rates in this study might be due to the high proportion of patients treated with a Factor Xa inhibitor.

Platelet reactivity might vary according to the time of blood draw and medication intake [23]. Considering that in this study all patients similarly received clopidogrel maintance dose 1- 2 h before blood draw, it does not seem plausible that time of measurement from PCI might be a strong reason for the high HPR rates.

TEG values differed deepending on external factors but the univariable analysis suggested that peri-procedural treatment with OAC and an ACS were not associated with both of the clinical outcomes. In this context the predictive value of these factors remains only limited and we attibuite the lack of association to the small sample size and the potential differences in the cut-offs for this patient cohort.

### Prognostic Implications of Platelet Aggregation Status and Platelet–Fibrin Cloth Strength

Numerous studies have established that HPR and LPR status assessed by TEG predict an increased ischemic or bleeding risk in patients with CAD undergoing PCI [6, 11, 20]. After elective stenting in CAD patients, MA_ADP_ had a good discriminative capacity for ischemic events with an area under the curve (AUC) of 0.84 [6]. Guided escalation of antiplatelet therapy in patients with LPR status assessed by TEG improved ischemic clinical outcomes without increasing bleeding risk in patients undergoing PCI [24]. Other studies suggest that the alteration of hemostatic status assessed by TEG was associated with increased thrombo-ischemic risk in patients with CAD. The risk for ischemic complications was increased with twofold in patients undergoing PCI procedure and with high MA_Thrombin_ levels [19]. Some data even suggests that clot-strength is more predictive than ADP-induced platelet aggregation on predicting ischemic events [20]. A consensus document indicates the use of assays such as TEG to better assess prognosis of patients undergoing PCI and guide antiplatelet therapy in specific clinical scenarios [16].

In our study HPR, LPR and MA_Thrombin_ were not associated with the clinical outcomes. This might be due to the low sample size or because for this specific patient cohort different TEG6s cut-off-values might be more appropriate. In fact, one study determined a different cut-off compared to the one suggested by the manufacturer for fibrin-clot strength [25]. Consistently with the trend in association of platelet–fibrin clot strength with the composite ischemic outcome in our study, previous data showed that the viscoelastic properties of platelet–fibrin clot strength might be an equally important risk factor as HPR status for ischemic events after PCI [26].

While recent studies suggest that this aggregation-coagulation pattern associates with increased risk of MACE and all-cause mortality in patients undergoing PCI without AF [11], this combined pattern assessed by TEG might be a better marker to help improve risk stratification for personalized therapy also in patients with AF who are treated with an OAC after PCI.

### Limitations of the Study

This is an observational study and the findings should be considered as only hypothesis generating. Furthermore, the small sample size and the lower than expected event rates limit the determination of any association with the clinical outcomes. Platelet aggregation and hemostasis may vary over time [27] and we performed only one single measurement at day 1–3 after PCI. The lack of serial TEG measurements, especially the missing baseline status of the patients, is another limitation of the current study. The absence of baseline measurements makes it difficult to assess changes in hemostatic parameters after PCI, which in turn limits the generalizability of our findings to broader patient populations.

To our knowledge this is the first study to assess the platelet aggregation and haemostatic profiles of a cohort of individuals with AF, treated with an OAC and undergoing PCI.

This study was not designed to determine cut-offs of TEG for this patient cohort, but further studies with larger cohorts are necessary to clarify the appropriate cut-offs and better evaluate the prognostic impact of TEG in patients with AF undergoing PCI.

## Conclusions

The prevalence of HPR status in patients with AF undergoing PCI treated with clopidogrel and OAC is high (~ 60%). However, neither HPR, nor LPR predicted MACE and bleeding risk in this specific patient population. Nevertheless, patients with both HPR status and increased platelet–fibrin clot strength may have a higher risk of the primary composite ischemic outcome.

This study was underpowered to detect a significant association with the clinical outcomes, but it suggests that other cut-offs may be more appropriate for this cohort of patients. Future studies with larger sample sizes and refined TEG cut-off values may help to better define its role in guiding personalized antithrombotic strategies for this high-risk cohort.

## Supplementary Information

Below is the link to the electronic supplementary material.

Supplementary file1 (DOCX 2.38 MB)

## Data Availability

Anonymised data could be available upon reasonable request.
