# Supplementary material for: Platelet Reactivity and Fibrin Clot-Strength as assessed by TEG in Patients with Atrial Fibrillation undergoing Percutaneous Coronary Intervention
Source: J Cardiovasc Transl Res. 2025 Aug 27;18(5):1312–24. doi: 10.1007/s12265-025-10673-4 (PMC12630191; doi:10.1007/s12265-025-10673-4)
Supplement: Supplementary file 1 — Supplementary file1 (DOCX 2.38 MB) [file 12265_2025_10673_MOESM1_ESM.docx]

# **SUPPLEMENT**

## **Table S1. Key inclusion and exclusion criteria**

| **Inclusion criteria** |
| --- |
| - Informed consent - ≥18 years of age or older - Atrial fibrillation with an indication for oral anticoagulation (i.e. CHA2DS2VASC score ≥1 for males, ≥2 for females) - Percutaneous coronary intervention within 3 days prior to enrolment |
| **Exclusion criteria** |
| - Contraindication to a DOAC (i.e. apixaban, dabigatran, edoxaban, rivaroxaban) or clopidogrel - History of stent-thrombosis - Uncompliant patient from the point of view of the principal investigator like for example: assumed non-compliance, frequent use of alcohol and drugs or not willing to proceed according to the protocol (patient not willing to follow-up) - GPIIb/IIa inhibitor in the last 24h - Use of prasugrel or ticagrelor in the last 7 days |

## **Table S2. Periprocedural medication, medication at discharge and at 6 months follow-up.**

| **Medication** | **Total**  **N=** **168** | | |
| --- | --- | --- | --- |
| **Periprocedural medication** |  | |  |
| ASA | 157 | | (93%) |
| ≤ 300 mg loading | 68 | (40%) | |
| ≥ 300 mg loading | 70 | (42%) | |
| Maintenance therapy | 19 | (11%) | |
| Clopidogrel | 167 | (99%) | |
| 300 mg loading | 33 | (20%) | |
| 600 mg loading | 100 | (60%) | |
| Maintenance therapy | 34 | (20%) | |
| OAC bridging therapy | 17 | (10%) | |
| OAC at time of measurement | 138 | (82%) | |
| **Medication at discharge** |  |  | |
| ASA at discharge | 39 | (23%) | |
| Clopidogrel at discharge | 168 | (100%) | |
| OAC at discharge | 167 | (99%) | |
| Vitamin K antagonist | 7 | (4%) | |
| Edoxaban | 37 | (22%) | |
| Apixaban | 59 | (35%) | |
| Rivaroxaban | 59 | (35%) | |
| Dabigatran | 5 | (3%) | |
| **Medication at 6 months follow-up** |  |  | |
| Clopidogrel at follow-up | 124 | (74%) | |
| OAC at follow-up | 158 | (94%) | |
| Values are n (%). Abbreviations: ASA, acetylsalicylic acid; OAC, oral anticoagulation. | | | |
|  | | | |

## **Table S3. Association of HPR status when considering % aggregation APD (% Agg. ADP ≥ 83%) with the primary and secondary outcomes at 6 months ± 2 weeks follow-up.**

| **Outcomes** | **Total**  **n=168** | | **Agg.**  **ADP≥ 83%**  **n=46**  **(27%)** | **Agg. ADP<83%**  **n=122**  **(73%)** | **p-Value** |  |
| --- | --- | --- | --- | --- | --- | --- |
| **Primary outcomes** |  |  |  |  |  |  |
| MACE | 17 | (10%) | 5 (11%) | 12 (10%) | 0.704 |  |
| Death | 10 | (6%) | 2 (4%) | 8 (7%) | 0.582 |  |
| Myocardial infarction | 5 | (3%) | 2 (4%) | 3 (2%) | 0.527 |  |
| Stroke | 2 | (1%) | 1 (2%) | 1 (1%) | 0.476 |  |
| **Secondary outcomes** |  |  |  |  |  |  |
| NMCR or major | 17 | (10%) | 9 (20%) | 15 (12%) | 0.238 |  |
| NMCR | 9 | (5%) | 3 (7%) | 6 (5%) | 0.689 |  |
| Major | 8 | (5%) | 5 (11%) | 10 (8%) | 0.599 |  |
| Any bleedings | 69 | (41%) | 21 (46%) | 51 (42%) | 0.683 |  |
| Minor bleedings | 52 | (31%) | 12 (26%) | 36 (30%) | 0.640 |  |
| The values are in absolute number and percentage, n (%). Abbreviations: MACE major adverse cardiac events, NMCR non-major clinically relevant, HPR high platelet reactivity. | | | | | | |

## **Table S4. Association of baseline characteristics with MA_Thrombin_ and MA_ADP_.**

| **Independent variables** | **MA_Thrombin_** | | **MA_ADP_** | |
| --- | --- | --- | --- | --- |
|  | **Unstandardized B- coefficient (95%CI)** | **p-value** | **Unstandardized B- coefficient (95%CI)** | **p-value** |
| Age | 0.14 (0.02-0.26) | 0.021 | -0.02 (-0.37-0.33) | 0.909 |
| Sex | 2.99 (0.67-5.32) | 0.012 | 3.25 (-3.28-9.78) | 0.326 |
| BMI, kg/m2 | 0.04 (-0.17-0.24) | 0.716 | 0.45 (-0.16-1.06) | 0.144 |
| Type of AF (persistent vs. paroxysmal) | 0.96 (-0.25-2.17) | 0.120 | -1.17 (-4.56-2.23) | 0.498 |
| Renal impairment | 0.62 (-1.70-2.94) | 0.599 | -3.83 (-10.3-2.67) | 0.247 |
| ACS | 2.21 (0.28-4.15) | 0.025 | 5.90 (0.49-11.35) | 0.033 |
| Interruption of OAC | 2.85 (0.72-4.98) | 0.009 | 4.81 (-.113-10.7) | 0.112 |
| Thrombocyte count, x10³/μL | 0.021 (0.01-0.03) | <0.001 | 0.04 (0.01-0.07) | 0.016 |
| Fibrinogen, mg/dl | 0.01 (0.00-0.02) | 0.055 | 0.22 (0.01-0.06) | 0.024 |
| IPF, % | 0.25 (-0.11-0.60) | 0.176 | 0.35 (-0.72-1.42) | 0.515 |

| The values are in absolute number. Abbreviations: CI confidence interval, BMI body mass index, AF atrial fibrillation, IPF immature platelet function |
| --- |

## **Figure S1. Kaplan-Meier-curves for the primary composite ischemic outcome MACE at 6 months for patients (A) with HPR vs. no HPR and for the secondary bleeding outcome for patients with (B) LPR vs. no LPR.**

**(A)**

**
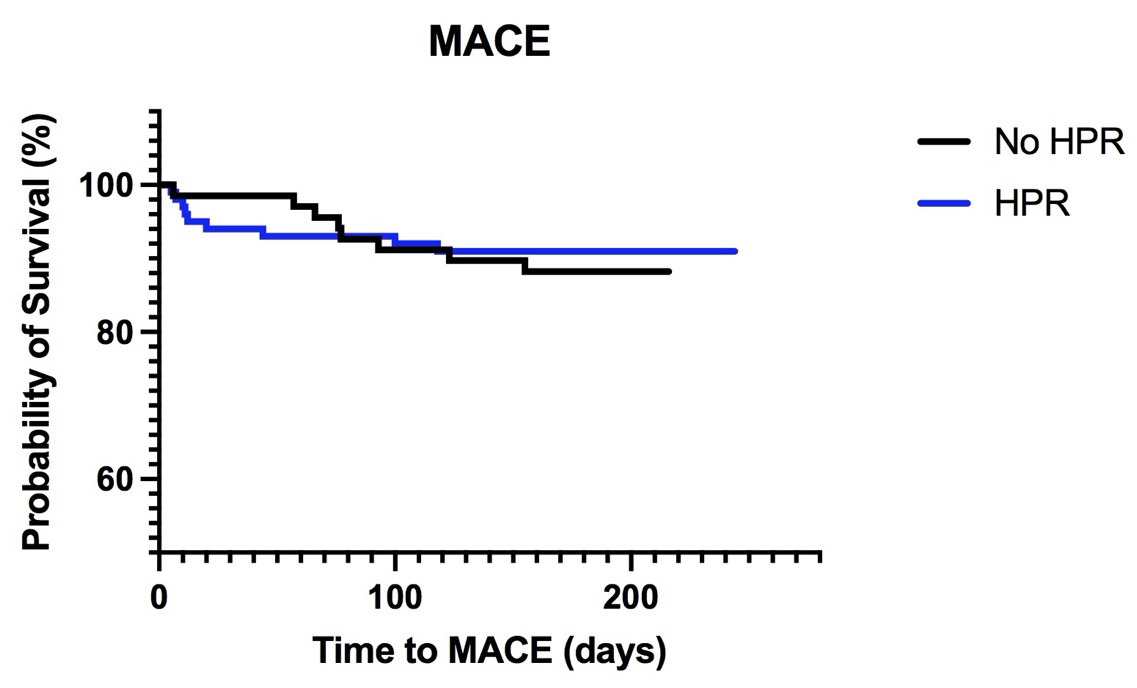
**

**(B)**

**
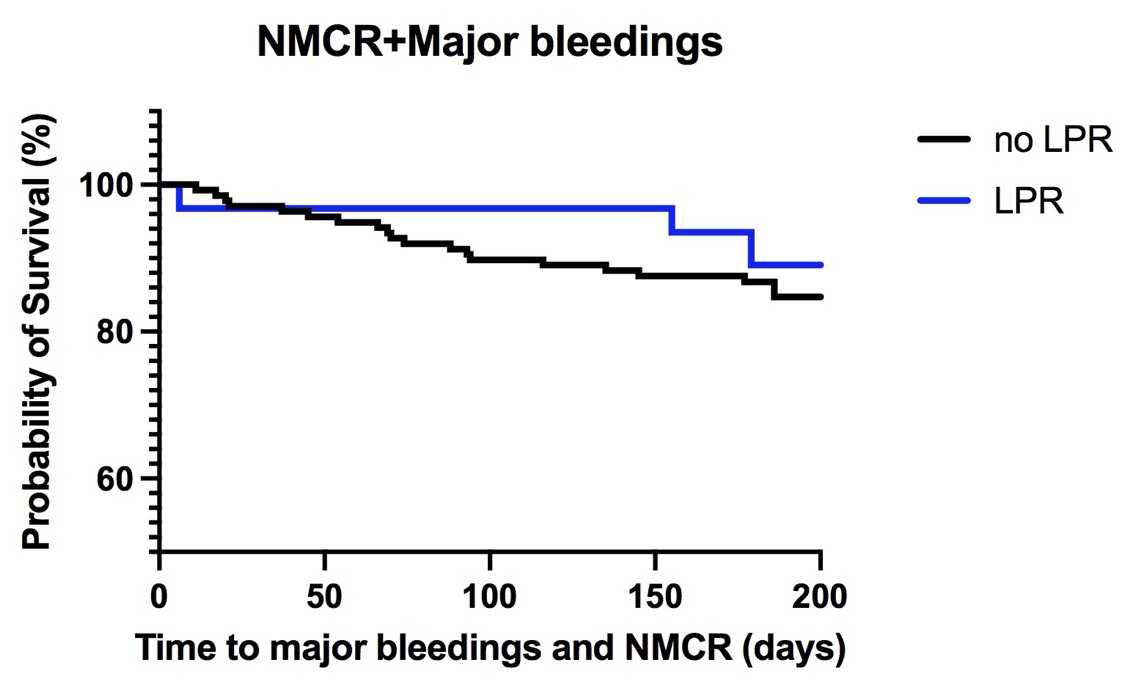
**

## **Figure S2. Distribution of values from platelet mapping assay as follows: (A) %-Agg. ADP and (B) MA_ASA_. Black lines represent median and interquartile range. Red lines indicate reference values suggested by the manufacturer.**

**(A)**

**(B)**

## **Figure S3. Distribution of values from the global hemostasis assay as follows: (A) MA_CRT_, (B) MA_CFF_, (C) CRT-ly30 and (D) CK-R. Black lines represent median and interquartile range. Red lines indicate reference values suggested by the manufacturer**.


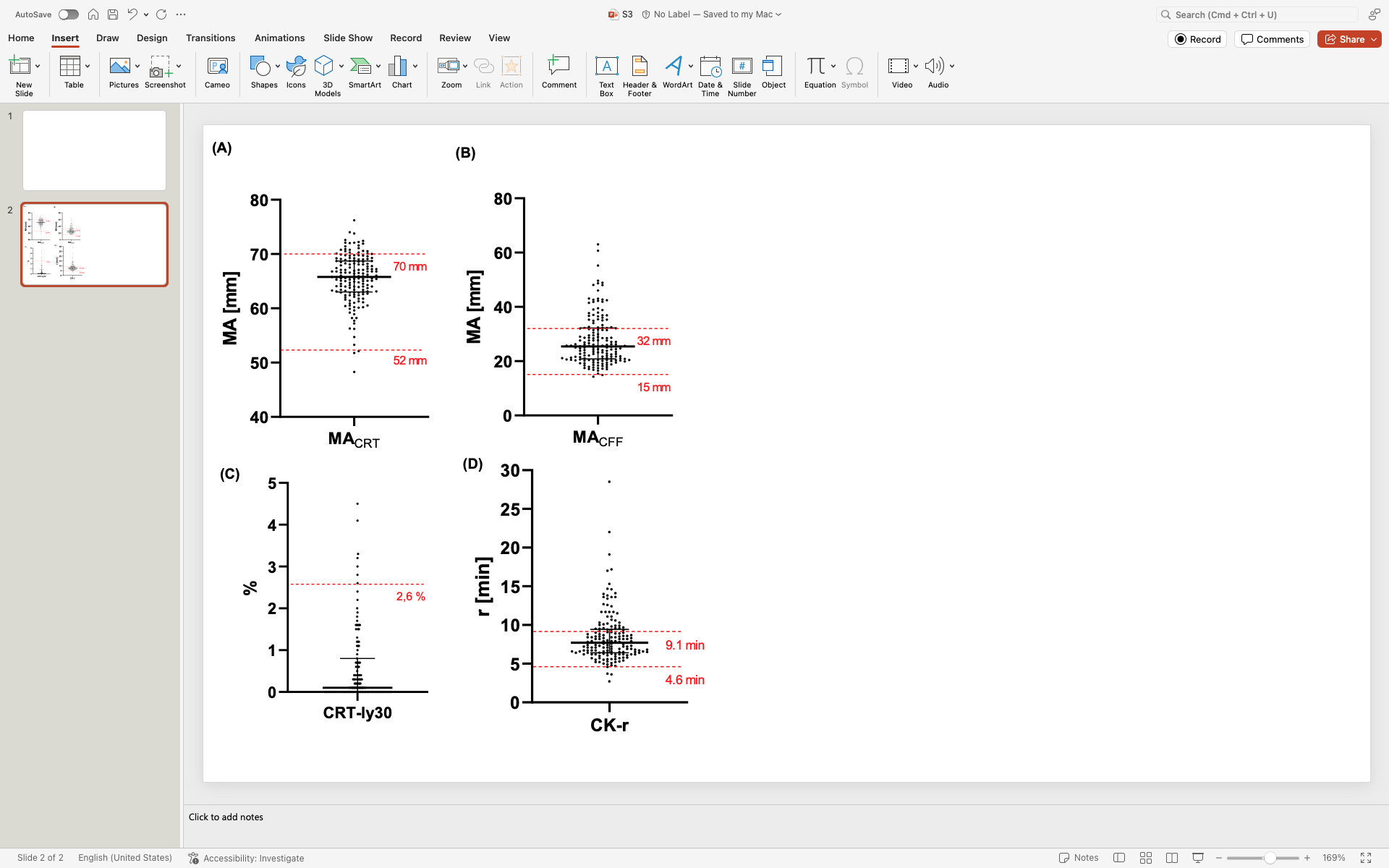


**Figure S4. Distribution of overall aggregation values and on-clopidogrel platelet reactivity represented respectively by (A)MA_Thrombin_ and (B) MA_ADP_ according to the day of TEG measurement after PCI. Red lines represent median.**

**(A)**

**
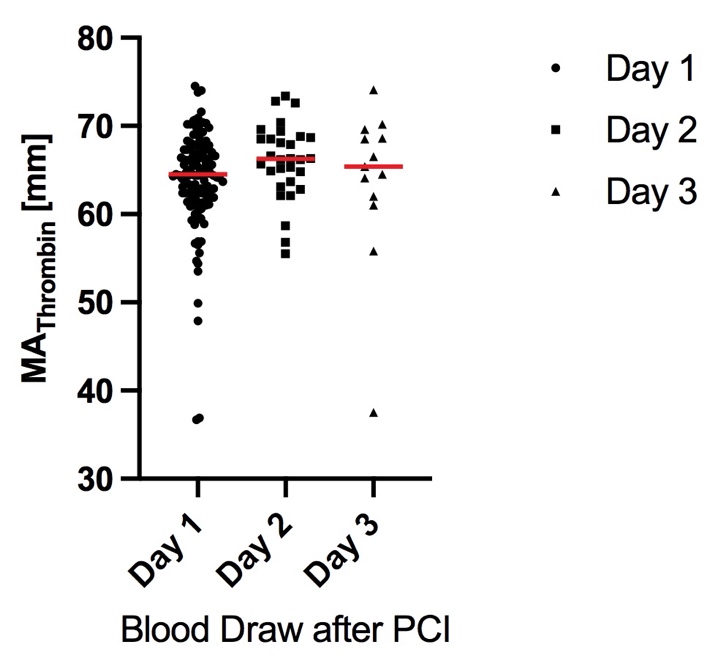
**

**(B)**

**
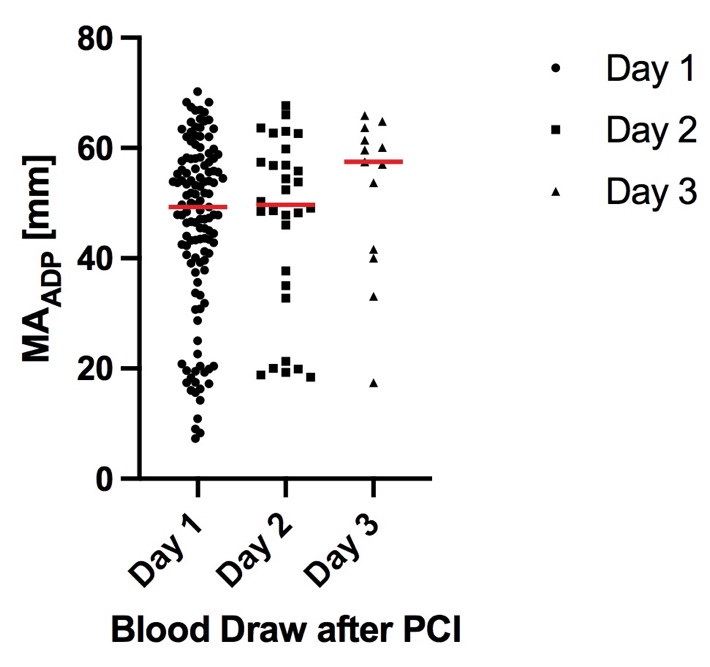
**

**Figure S5. Distribution of on-clopidogrel platelet reactivity (MA_ADP_) according to the (A) indication for PCI and (B) interruption of OAC.**

**(A)**

**
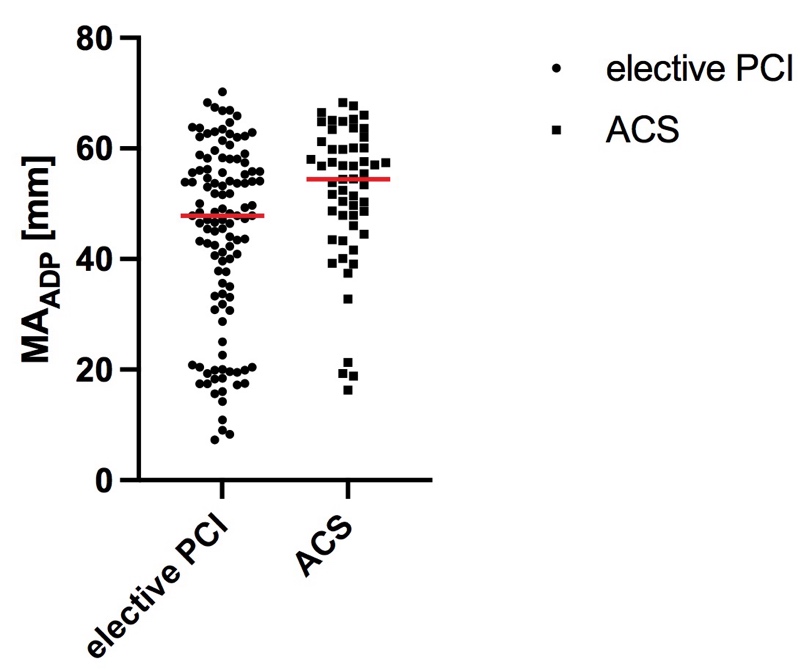
**

**(B)**

**
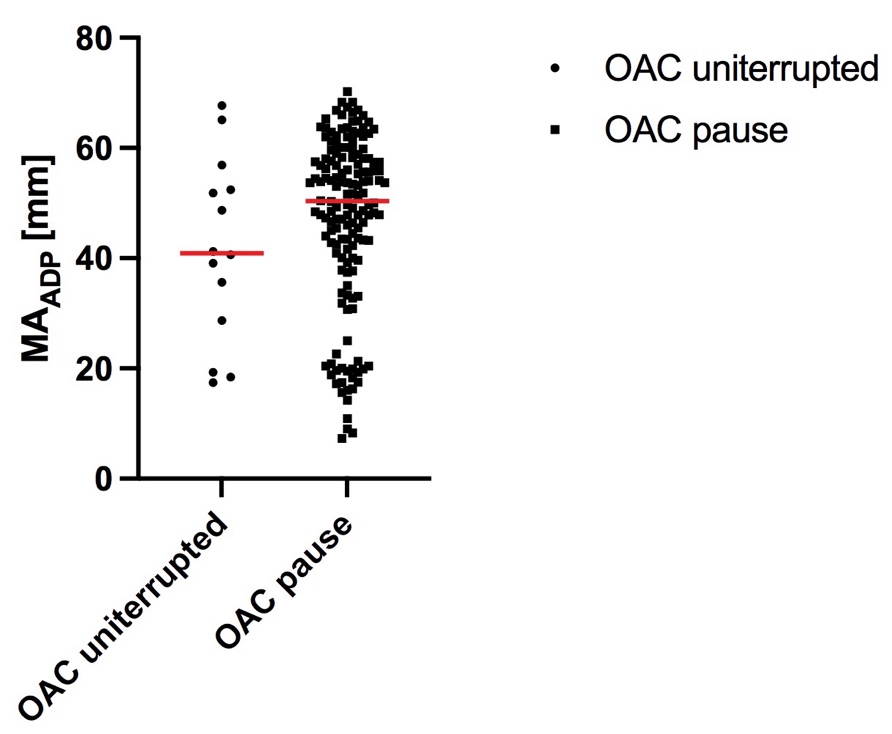
**

## **Figure S6. Correlation of MA_Thrombin_ and MA_ADP_ with the thrombocyte count (A, B) and fibrinogen (C, D). The red line represents the overall trend of correlation between the two variables.**

**
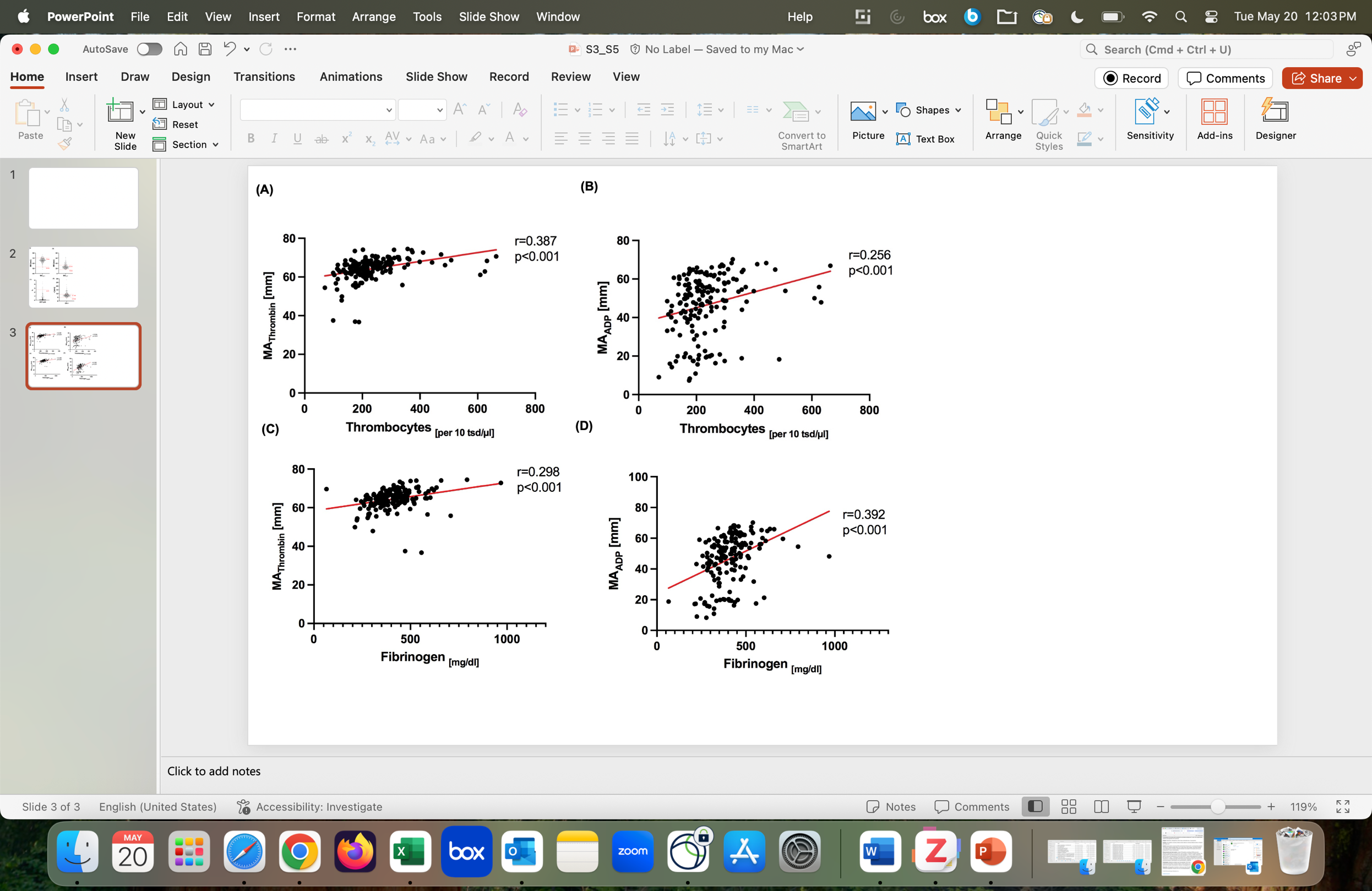
**

## **Figure S7. Correlation of MA_ADP_ with R time as assessed by TEG. The red line represents the overall trend of correlation between the two variables.**

**
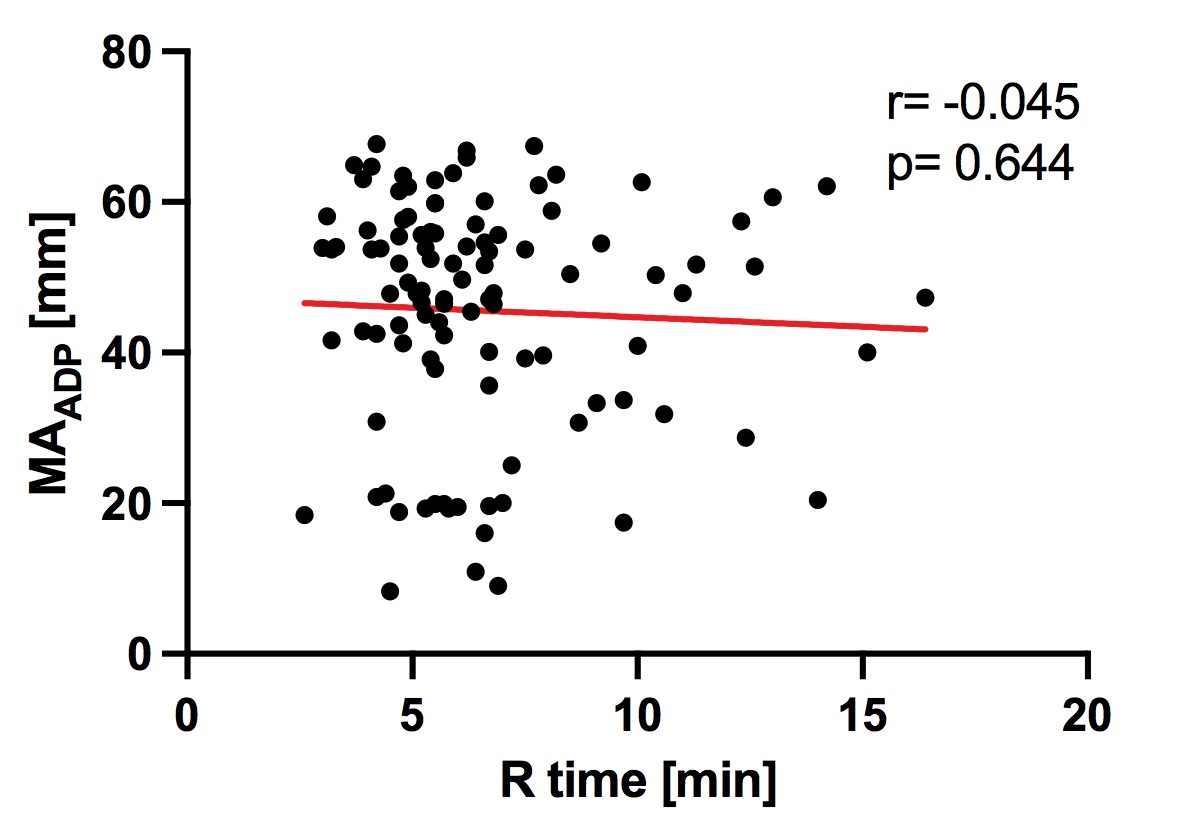
**
